# Supplementary figures and images for: A rapid spread of the stony coral tissue loss disease outbreak in the Mexican Caribbean
Source: PeerJ. 2019 Nov 26;7:e8069. doi: 10.7717/peerj.8069 (PMC6883952; doi:10.7717/peerj.8069)

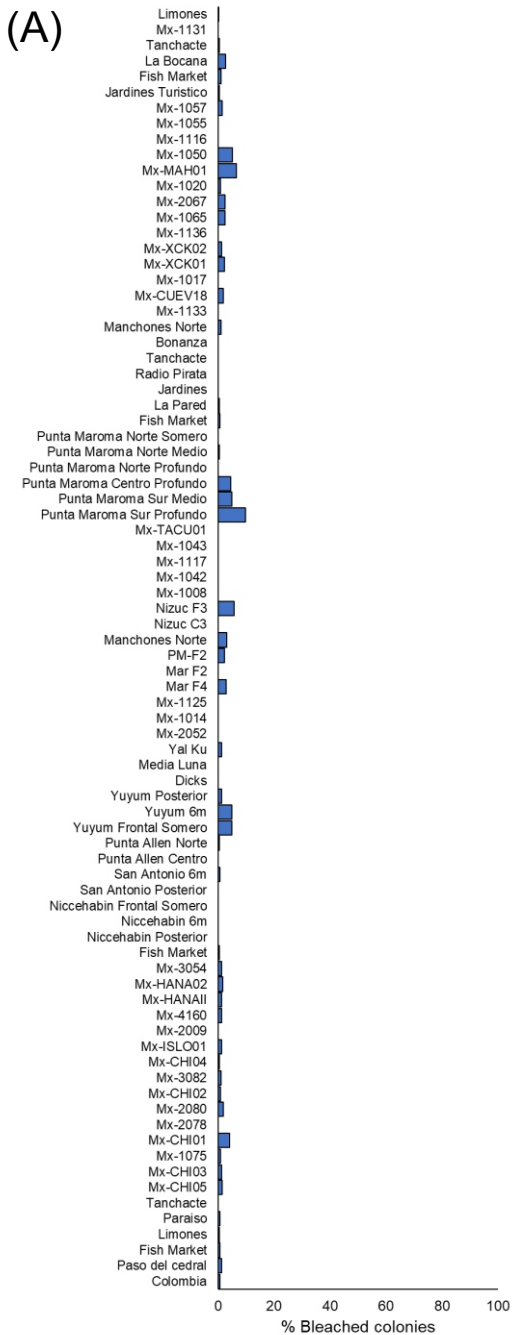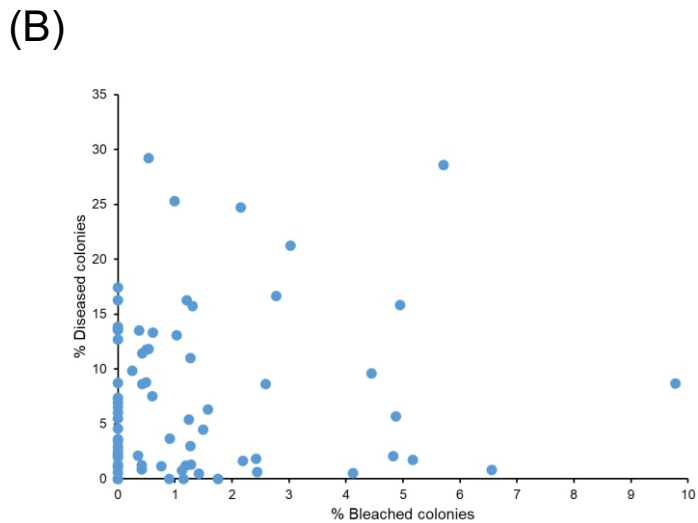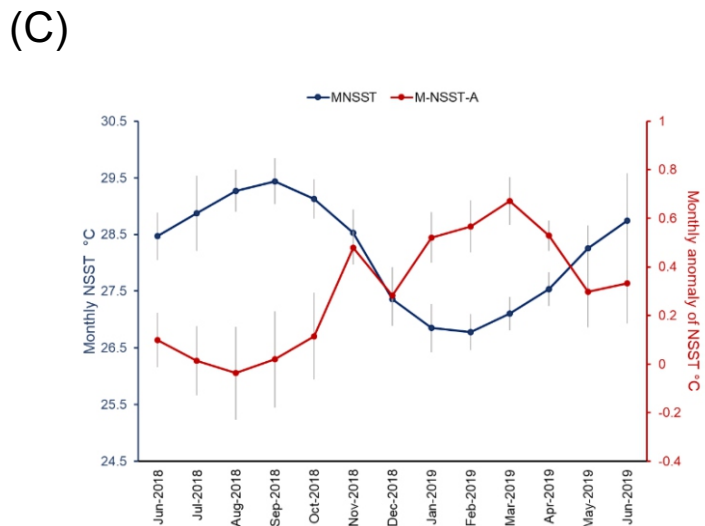

Supplement: Supplemental Information 1 — (A) Percentage of bleached colonies across the 82 surveyed reef sites (ordered by surveyed date). (B) Relationship between the percentage of bleached colonies and the stony coral tissue loss disease prevalence for the 82 surveyed sites (y = 0.43 x + 6.58, R² = 0.01). (C) Average monthly nighttime Sea Surface Temperature (MNSST) in blue (± St. Dev) and average monthly nighttime Sea Surface Temperature Anomaly (MNSST-A) in red (± St. Dev) from nine localities across the region (Cancun, Puerto Morelos, Cozumel East coast, Cozumel West coast, Tulum, Punta Allen, Mahahual, Xcalak and Chinchorro Bank). For (A) and (B) coral colonies completely and partially bleached were considered. For (C) the information was obtained from the Marine-Coastal Information and Analysis System of CONABIO (https://simar.conabio.gob.mx/), which use data from the following sources: Group for High Resolution Sea Surface Temperature (GHRSST), Jet Propulsion Laboratory (JPL), Physical Oceanography Distributed Active Archive Center (PO.DAAC), National Aeronautics and Space Administration (NASA). References: Cerdeira-Estrada S, Martell-Dubois R, Valdéz J, Ressl R. 2019. Monthly nighttime Sea Surface Temperature Anomaly (M-NSST-A) at 1-km. Satellite-based ocean monitoring system (SATMO). Marine-Coastal Information and Analysis System (SIMAR). CONABIO. México. Dataset accessed (2018-05-10) at simar.conabio.gob.mx. GHRSST-MUR (1jun2002-realtime): JPL MUR MEaSUREs Project. 2015. GHRSST Level 4 MUR Global Foundation Sea Surface Temperature Analysis (v4.1). Ver. 4.1. PO.DAAC, CA, USA. Dataset accessed. [file peerj-07-8069-s001.pdf]
