# Supplementary material for: A rapid spread of the stony coral tissue loss disease outbreak in the Mexican Caribbean
Source: PeerJ. 2019 Nov 26;7:e8069. doi: 10.7717/peerj.8069 (PMC6883952; doi:10.7717/peerj.8069)
Supplement: Supplemental Information 2 — Susceptible species are those that presented more than 10% of SCTLD (Fig. 2; Table S2). Parque Nacional Arrecifes de Cozumel = PNAC; Parque Nacional Arrecife de Puerto Morelos = PNAPM; Biodiversity and Reef Conservation Lab, UNAM = Barco-Lab; Healthy Reefs Initiative = HRI. [file peerj-07-8069-s002.docx]

**Table S1. Monitoring data by site and year on coral diseases from 2005 to 2019. Susceptible species are those that presented more than 10% of SCTLD (Fig. 2, Table S2). Parque Nacional Arrecifes de Cozumel = PNAC; Parque Nacional Arrecife de Puerto Morelos = PNAPM; Biodiversity and Reef Conservation Lab, UNAM = Barco-Lab; Healthy Reefs Initiative = HRI.**

| **Institution** | **Year** | **Time period (as in Fig.4)** | **Site name** | **# Transects** | **# Coral species** | **Total number of surveyed coral colonies** | **%Total diseased colonies** | **# of colonies of susceptible species** | **% diseased colonies of susceptible species** | **Dead colonies of susceptible species** |
| --- | --- | --- | --- | --- | --- | --- | --- | --- | --- | --- |
| PNAC | 2005 | 2005/2006 | Buenavista | 6 | 10 | 60 | 0.0 | 25 | 0.0 | 0 |
| PNAC | 2005 | 2005/2006 | Castillo | 6 | 7 | 51 | 0.0 | 28 | 0.0 | 0 |
| PNAC | 2005 | 2005/2006 | Chankanaab | 6 | 12 | 107 | 2.8 | 44 | 0.9 | 0 |
| PNAC | 2005 | 2005/2006 | Colombia | 6 | 14 | 125 | 0.8 | 40 | 0.0 | 0 |
| PNAC | 2005 | 2005/2006 | Dalila | 6 | 16 | 124 | 1.6 | 37 | 1.6 | 0 |
| PNAC | 2005 | 2005/2006 | Hanan | 5 | 12 | 43 | 0.0 | 21 | 0.0 | 0 |
| PNAC | 2005 | 2005/2006 | Hanan II | 6 | 11 | 62 | 0.0 | 34 | 0.0 | 0 |
| PNAC | 2005 | 2005/2006 | Islote | 6 | 11 | 57 | 0.0 | 17 | 0.0 | 0 |
| PNAPM | 2005 | 2005/2006 | Limones | 3 | 10 | 86 | 11.6 | 62 | 10.5 | 0 |
| HRI | 2005 | 2005/2006 | Mx-1005 | 5 | 11 | 37 | 8.1 | 14 | 2.7 | 0 |
| HRI | 2005 | 2005/2006 | Mx-1006 | 4 | 16 | 49 | 8.2 | 9 | 0.0 | 0 |
| HRI | 2005 | 2005/2006 | Mx-1008 | 4 | 12 | 55 | 1.8 | 20 | 0.0 | 0 |
| HRI | 2005 | 2005/2006 | Mx-1010 | 5 | 12 | 46 | 0.0 | 16 | 0.0 | 0 |
| HRI | 2005 | 2005/2006 | Mx-1011 | 2 | 2 | 2 | 0.0 | 2 | 0.0 | 0 |
| HRI | 2005 | 2005/2006 | Mx-1012 | 5 | 17 | 63 | 0.0 | 43 | 0.0 | 0 |
| HRI | 2005 | 2005/2006 | Mx-1014 | 4 | 12 | 42 | 2.4 | 25 | 2.4 | 0 |
| HRI | 2005 | 2005/2006 | Mx-1035 | 4 | 6 | 8 | 0.0 | 5 | 0.0 | 0 |
| HRI | 2005 | 2005/2006 | Mx-1036 | 4 | 7 | 20 | 0.0 | 6 | 0.0 | 0 |
| HRI | 2005 | 2005/2006 | Mx-1037 | 4 | 15 | 39 | 0.0 | 15 | 0.0 | 0 |
| HRI | 2005 | 2005/2006 | Mx-1040 | 4 | 9 | 31 | 0.0 | 11 | 0.0 | 0 |
| HRI | 2005 | 2005/2006 | Mx-1042 | 4 | 10 | 47 | 0.0 | 24 | 0.0 | 0 |
| HRI | 2005 | 2005/2006 | Mx-1043 | 4 | 13 | 45 | 0.0 | 22 | 0.0 | 0 |
| HRI | 2005 | 2005/2006 | Mx-1044 | 3 | 5 | 11 | 0.0 | 6 | 0.0 | 0 |
| HRI | 2005 | 2005/2006 | Mx-1045 | 3 | 14 | 22 | 0.0 | 9 | 0.0 | 0 |
| HRI | 2005 | 2005/2006 | Mx-1047 | 2 | 10 | 19 | 5.3 | 7 | 5.3 | 0 |
| HRI | 2005 | 2005/2006 | Mx-1048 | 2 | 10 | 30 | 0.0 | 6 | 0.0 | 0 |
| HRI | 2005 | 2005/2006 | Mx-1050 | 4 | 12 | 35 | 0.0 | 11 | 0.0 | 0 |
| HRI | 2005 | 2005/2006 | Mx-1053 | 4 | 14 | 32 | 0.0 | 14 | 0.0 | 0 |
| HRI | 2005 | 2005/2006 | Mx-1055 | 4 | 8 | 32 | 0.0 | 12 | 0.0 | 0 |
| HRI | 2005 | 2005/2006 | Mx-1057 | 4 | 8 | 13 | 0.0 | 5 | 0.0 | 0 |
| HRI | 2005 | 2005/2006 | Mx-1060 | 3 | 3 | 15 | 0.0 | 14 | 0.0 | 0 |
| HRI | 2005 | 2005/2006 | Mx-1061 | 3 | 6 | 13 | 0.0 | 12 | 0.0 | 0 |
| HRI | 2005 | 2005/2006 | Mx-1066 | 2 | 5 | 6 | 16.7 | 4 | 16.7 | 0 |
| HRI | 2005 | 2005/2006 | Mx-1112 | 4 | 9 | 20 | 0.0 | 4 | 0.0 | 0 |
| HRI | 2005 | 2005/2006 | Mx-1116 | 4 | 9 | 33 | 0.0 | 24 | 0.0 | 0 |
| HRI | 2005 | 2005/2006 | Mx-1117 | 3 | 7 | 33 | 0.0 | 3 | 0.0 | 0 |
| HRI | 2005 | 2005/2006 | Mx-1125 | 5 | 8 | 39 | 0.0 | 4 | 0.0 | 0 |
| HRI | 2005 | 2005/2006 | Mx-1126 | 4 | 5 | 27 | 0.0 | 1 | 0.0 | 0 |
| HRI | 2005 | 2005/2006 | Mx-1131 | 3 | 8 | 24 | 0.0 | 14 | 0.0 | 0 |
| HRI | 2005 | 2005/2006 | Mx-1134 | 4 | 10 | 41 | 0.0 | 3 | 0.0 | 0 |
| HRI | 2005 | 2005/2006 | Mx-2002 | 3 | 4 | 6 | 0.0 | 5 | 0.0 | 0 |
| HRI | 2005 | 2005/2006 | Mx-2007 | 4 | 18 | 59 | 1.7 | 18 | 1.7 | 0 |
| HRI | 2005 | 2005/2006 | Mx-2009 | 3 | 6 | 28 | 0.0 | 1 | 0.0 | 0 |
| HRI | 2005 | 2005/2006 | Mx-2044 | 3 | 14 | 75 | 4.0 | 15 | 4.0 | 0 |
| HRI | 2005 | 2005/2006 | Mx-2052 | 5 | 17 | 74 | 1.4 | 48 | 1.4 | 0 |
| HRI | 2005 | 2005/2006 | Mx-2110 | 4 | 7 | 35 | 0.0 | 1 | 0.0 | 0 |
| HRI | 2005 | 2005/2006 | Mx-2113 | 2 | 2 | 2 | 0.0 | 1 | 0.0 | 0 |
| HRI | 2005 | 2005/2006 | Mx-2127 | 3 | 5 | 9 | 0.0 | 2 | 0.0 | 0 |
| HRI | 2005 | 2005/2006 | Mx-3009 | 3 | 12 | 64 | 0.0 | 5 | 0.0 | 0 |
| HRI | 2005 | 2005/2006 | Mx-3039 | 4 | 5 | 14 | 0.0 | 5 | 0.0 | 0 |
| HRI | 2005 | 2005/2006 | Mx-3048 | 3 | 10 | 28 | 0.0 | 8 | 0.0 | 0 |
| HRI | 2005 | 2005/2006 | Mx-3054 | 4 | 14 | 50 | 0.0 | 19 | 0.0 | 0 |
| HRI | 2005 | 2005/2006 | Mx-3117 | 4 | 4 | 41 | 0.0 | 1 | 0.0 | 0 |
| HRI | 2005 | 2005/2006 | Mx-4156 | 3 | 5 | 13 | 0.0 | 4 | 0.0 | 0 |
| HRI | 2005 | 2005/2006 | Mx-4158 | 4 | 10 | 38 | 0.0 | 9 | 0.0 | 0 |
| HRI | 2005 | 2005/2006 | Mx-4159 | 4 | 7 | 19 | 0.0 | 4 | 0.0 | 0 |
| HRI | 2005 | 2005/2006 | Mx-4160 | 4 | 12 | 37 | 0.0 | 15 | 0.0 | 0 |
| PNAC | 2005 | 2005/2006 | Paraiso | 6 | 17 | 109 | 0.9 | 50 | 0.9 | 0 |
| PNAC | 2005 | 2005/2006 | Paso del Cedral | 6 | 18 | 127 | 0.8 | 29 | 0.8 | 0 |
| PNAPM | 2005 | 2005/2006 | Puerto Morelos | 2 | 5 | 31 | 0.0 | 31 | 0.0 | 0 |
| PNAC | 2005 | 2005/2006 | Villablanca | 6 | 11 | 36 | 0.0 | 20 | 0.0 | 0 |
| PNAC | 2005 | 2005/2006 | Yucab | 6 | 14 | 110 | 0.0 | 36 | 0.0 | 0 |
| PNAPM | 2006 | 2005/2006 | Bonanza | 6 | 10 | 72 | 0.0 | 57 | 0.0 | 0 |
| PNAPM | 2006 | 2005/2006 | Jardines | 2 | 9 | 77 | 1.3 | 59 | 1.3 | 0 |
| HRI | 2006 | 2005/2006 | Mx-1019 | 2 | 4 | 5 | 0.0 | 1 | 0.0 | 0 |
| HRI | 2006 | 2005/2006 | Mx-1020 | 3 | 7 | 17 | 0.0 | 8 | 0.0 | 0 |
| HRI | 2006 | 2005/2006 | Mx-1026 | 5 | 14 | 56 | 0.0 | 27 | 0.0 | 0 |
| HRI | 2006 | 2005/2006 | Mx-1027 | 4 | 11 | 18 | 0.0 | 11 | 0.0 | 0 |
| HRI | 2006 | 2005/2006 | Mx-1028 | 4 | 11 | 36 | 0.0 | 12 | 0.0 | 0 |
| HRI | 2006 | 2005/2006 | Mx-1029 | 2 | 7 | 15 | 0.0 | 5 | 0.0 | 0 |
| HRI | 2006 | 2005/2006 | Mx-1032 | 4 | 11 | 33 | 0.0 | 8 | 0.0 | 0 |
| HRI | 2006 | 2005/2006 | Mx-1034 | 4 | 4 | 8 | 0.0 | 4 | 0.0 | 0 |
| HRI | 2006 | 2005/2006 | Mx-1059 | 4 | 15 | 40 | 5.0 | 10 | 0.0 | 0 |
| HRI | 2006 | 2005/2006 | Mx-1062 | 4 | 8 | 22 | 0.0 | 14 | 0.0 | 0 |
| HRI | 2006 | 2005/2006 | Mx-1065 | 4 | 9 | 36 | 0.0 | 8 | 0.0 | 0 |
| HRI | 2006 | 2005/2006 | Mx-1092 | 4 | 7 | 13 | 0.0 | 1 | 0.0 | 0 |
| HRI | 2006 | 2005/2006 | Mx-1097 | 4 | 6 | 21 | 0.0 | 1 | 0.0 | 0 |
| HRI | 2006 | 2005/2006 | Mx-1103 | 4 | 5 | 25 | 0.0 | 1 | 0.0 | 0 |
| HRI | 2006 | 2005/2006 | Mx-1107 | 4 | 7 | 12 | 0.0 | 3 | 0.0 | 0 |
| HRI | 2006 | 2005/2006 | Mx-1109 | 4 | 9 | 52 | 0.0 | 9 | 0.0 | 0 |
| HRI | 2006 | 2005/2006 | Mx-1136 | 4 | 8 | 27 | 0.0 | 6 | 0.0 | 0 |
| HRI | 2006 | 2005/2006 | Mx-2024 | 3 | 2 | 3 | 0.0 | 3 | 0.0 | 0 |
| HRI | 2006 | 2005/2006 | Mx-2025 | 4 | 11 | 34 | 0.0 | 16 | 0.0 | 0 |
| HRI | 2006 | 2005/2006 | Mx-2033 | 4 | 10 | 42 | 0.0 | 17 | 0.0 | 0 |
| HRI | 2006 | 2005/2006 | Mx-2063 | 4 | 3 | 9 | 0.0 | 6 | 0.0 | 0 |
| HRI | 2006 | 2005/2006 | Mx-2067 | 4 | 12 | 40 | 2.5 | 18 | 2.5 | 0 |
| HRI | 2006 | 2005/2006 | Mx-2088 | 4 | 7 | 17 | 0.0 | 4 | 0.0 | 0 |
| HRI | 2006 | 2005/2006 | Mx-2100 | 4 | 6 | 16 | 0.0 | 3 | 0.0 | 0 |
| HRI | 2006 | 2005/2006 | Mx-2102 | 3 | 2 | 4 | 0.0 | 3 | 0.0 | 0 |
| HRI | 2006 | 2005/2006 | Mx-2104 | 4 | 10 | 22 | 0.0 | 5 | 0.0 | 0 |
| HRI | 2006 | 2005/2006 | Mx-3021 | 1 | 5 | 10 | 0.0 | 5 | 0.0 | 0 |
| HRI | 2006 | 2005/2006 | Mx-3030 | 3 | 8 | 23 | 0.0 | 9 | 0.0 | 0 |
| HRI | 2006 | 2005/2006 | Mx-3099 | 4 | 8 | 29 | 0.0 | 10 | 0.0 | 0 |
| HRI | 2006 | 2005/2006 | Mx-3101 | 4 | 7 | 17 | 0.0 | 6 | 0.0 | 0 |
| HRI | 2006 | 2005/2006 | Mx-3130 | 4 | 9 | 29 | 0.0 | 8 | 0.0 | 0 |
| PNAPM | 2006 | 2005/2006 | RadioPirataCarriquiry | 10 | 10 | 75 | 0.0 | 31 | 0.0 | 0 |
| PNAPM | 2006 | 2005/2006 | Tanchacte Norte | 2 | 8 | 62 | 9.7 | 45 | 8.1 | 0 |
| PNAC | 2009 | 2009 | Chankanaab | 7 | 16 | 102 | 5.9 | 36 | 2.9 | 0 |
| PNAC | 2009 | 2009 | Colombia | 6 | 13 | 102 | 2.0 | 28 | 2.0 | 0 |
| PNAC | 2009 | 2009 | Dalila | 6 | 15 | 126 | 3.2 | 28 | 0.8 | 0 |
| HRI | 2009 | 2009 | Mx-1017 | 6 | 9 | 49 | 8.2 | 5 | 4.1 | 0 |
| HRI | 2009 | 2009 | Mx-1043 | 6 | 15 | 68 | 1.5 | 42 | 1.5 | 0 |
| HRI | 2009 | 2009 | Mx-1048 | 6 | 12 | 44 | 4.5 | 17 | 2.3 | 0 |
| HRI | 2009 | 2009 | Mx-1053 | 6 | 13 | 42 | 4.8 | 33 | 4.8 | 0 |
| HRI | 2009 | 2009 | Mx-1065 | 5 | 15 | 64 | 0.0 | 27 | 0.0 | 0 |
| HRI | 2009 | 2009 | Mx-1117 | 5 | 6 | 21 | 19.0 | 4 | 4.8 | 0 |
| HRI | 2009 | 2009 | Mx-1132 | 6 | 5 | 40 | 10.0 | 1 | 2.5 | 0 |
| HRI | 2009 | 2009 | Mx-1133 | 5 | 8 | 14 | 7.1 | 8 | 7.1 | 0 |
| HRI | 2009 | 2009 | Mx-1136 | 6 | 8 | 34 | 0.0 | 17 | 0.0 | 0 |
| HRI | 2009 | 2009 | Mx-2067 | 6 | 16 | 91 | 2.2 | 37 | 1.1 | 0 |
| HRI | 2009 | 2009 | Mx-3009 | 4 | 8 | 46 | 0.0 | 4 | 0.0 | 0 |
| PNAC | 2009 | 2009 | Paraiso | 6 | 15 | 93 | 3.2 | 43 | 3.2 | 0 |
| PNAC | 2009 | 2009 | Paso del Cedral | 6 | 20 | 111 | 1.8 | 25 | 1.8 | 0 |
| PNAC | 2009 | 2009 | Yucab | 6 | 10 | 52 | 5.8 | 27 | 5.8 | 0 |
| PNAC | 2011 | 2011/2012 | Chankanaab | 6 | 19 | 155 | 12.9 | 61 | 7.1 | 0 |
| PNAC | 2011 | 2011/2012 | Colombia | 6 | 22 | 192 | 10.4 | 70 | 5.7 | 0 |
| PNAC | 2011 | 2011/2012 | Dalila | 6 | 22 | 136 | 7.4 | 48 | 5.1 | 0 |
| HRI | 2011 | 2011/2012 | Mx-1017 | 2 | 8 | 57 | 0.0 | 1 | 0.0 | 0 |
| HRI | 2011 | 2011/2012 | Mx-1043 | 1 | 11 | 29 | 6.9 | 8 | 3.4 | 0 |
| HRI | 2011 | 2011/2012 | Mx-1048 | 4 | 14 | 130 | 2.3 | 26 | 2.3 | 0 |
| HRI | 2011 | 2011/2012 | Mx-1053 | 3 | 12 | 69 | 1.4 | 35 | 1.4 | 0 |
| HRI | 2011 | 2011/2012 | Mx-1117 | 1 | 6 | 10 | 0.0 | 3 | 0.0 | 0 |
| HRI | 2011 | 2011/2012 | Mx-1133 | 3 | 5 | 11 | 0.0 | 3 | 0.0 | 0 |
| HRI | 2011 | 2011/2012 | Mx-3009 | 4 | 13 | 138 | 0.7 | 11 | 0.7 | 0 |
| HRI | 2011 | 2011/2012 | Mx-BP01 | 2 | 13 | 41 | 12.2 | 18 | 7.3 | 0 |
| HRI | 2011 | 2011/2012 | Mx-DR01 | 2 | 15 | 39 | 10.3 | 18 | 7.7 | 0 |
| HRI | 2011 | 2011/2012 | Mx-Las Cuevas Deep | 2 | 15 | 123 | 1.6 | 68 | 0.8 | 0 |
| PNAC | 2011 | 2011/2012 | Paraiso | 6 | 20 | 111 | 10.8 | 51 | 8.1 | 0 |
| PNAC | 2011 | 2011/2012 | Paso del Cedral | 6 | 19 | 137 | 1.5 | 39 | 0.7 | 0 |
| PNAC | 2011 | 2011/2012 | Yucab | 6 | 20 | 104 | 6.7 | 51 | 4.8 | 0 |
| PNAPM | 2012 | 2011/2012 | Bonanza | 5 | 8 | 67 | 0.0 | 61 | 0.0 | 0 |
| PNAPM | 2012 | 2011/2012 | Jardines | 5 | 9 | 89 | 0.0 | 77 | 0.0 | 0 |
| PNAPM | 2012 | 2011/2012 | LA BOCANA | 5 | 11 | 124 | 0.0 | 115 | 0.0 | 0 |
| PNAPM | 2012 | 2011/2012 | La Pared | 4 | 11 | 69 | 0.0 | 60 | 0.0 | 0 |
| PNAPM | 2012 | 2011/2012 | Limones | 5 | 12 | 132 | 0.0 | 49 | 0.0 | 0 |
| HRI | 2012 | 2011/2012 | Mx-1055 | 1 | 4 | 14 | 7.1 | 6 | 7.1 | 0 |
| HRI | 2012 | 2011/2012 | Mx-1057 | 1 | 4 | 42 | 0.0 | 2 | 0.0 | 0 |
| HRI | 2012 | 2011/2012 | Mx-1065 | 1 | 8 | 14 | 0.0 | 5 | 0.0 | 0 |
| HRI | 2012 | 2011/2012 | Mx-2067 | 1 | 9 | 20 | 5.0 | 8 | 5.0 | 0 |
| HRI | 2012 | 2011/2012 | Mx-Bak 1 | 4 | 13 | 118 | 6.8 | 28 | 5.1 | 0 |
| HRI | 2012 | 2011/2012 | Mx-Bak 2 | 3 | 15 | 144 | 8.3 | 40 | 5.6 | 0 |
| HRI | 2012 | 2011/2012 | Mx-Las Cuevas Shallow | 2 | 15 | 117 | 3.4 | 67 | 3.4 | 0 |
| HRI | 2012 | 2011/2012 | Mx-Las Redes Deep | 2 | 16 | 104 | 3.8 | 47 | 3.8 | 0 |
| HRI | 2012 | 2011/2012 | Mx-Las Redes Shallow | 2 | 21 | 129 | 9.3 | 36 | 7.0 | 0 |
| HRI | 2012 | 2011/2012 | Mx-XCK01 | 2 | 10 | 38 | 2.6 | 7 | 2.6 | 0 |
| HRI | 2012 | 2011/2012 | Mx-XCK02 | 3 | 8 | 20 | 0.0 | 6 | 0.0 | 0 |
| HRI | 2012 | 2011/2012 | Mx-Yalku Deep | 2 | 14 | 101 | 7.9 | 29 | 4.0 | 0 |
| HRI | 2012 | 2011/2012 | Mx-Yalku Shallow | 2 | 19 | 106 | 12.3 | 52 | 9.4 | 0 |
| HRI | 2012 | 2011/2012 | Mx-Yalkuito Deep | 2 | 15 | 100 | 8.0 | 34 | 6.0 | 0 |
| HRI | 2012 | 2011/2012 | Mx-Yalkuito Shallow | 2 | 20 | 151 | 4.6 | 30 | 4.6 | 0 |
| PNAPM | 2012 | 2011/2012 | Radio Pirata | 5 | 10 | 67 | 0.0 | 45 | 0.0 | 0 |
| PNAPM | 2012 | 2011/2012 | Tanchacte Norte | 5 | 11 | 139 | 0.0 | 95 | 0.0 | 0 |
| PNAPM | 2012 | 2011/2012 | TANCHACTE SUR | 4 | 9 | 59 | 0.0 | 51 | 0.0 | 0 |
| PNAPM | 2014 | 2014 | Bonanza | 5 | 8 | 105 | 3.8 | 100 | 3.8 | 0 |
| PNAC | 2014 | 2014 | Chankanaab | 6 | 6 | 45 | 2.2 | 19 | 2.2 | 0 |
| PNAC | 2014 | 2014 | Colombia | 6 | 11 | 43 | 0.0 | 11 | 0.0 | 0 |
| PNAC | 2014 | 2014 | Dalila | 6 | 8 | 44 | 0.0 | 2 | 0.0 | 0 |
| PNAPM | 2014 | 2014 | Jardines | 5 | 13 | 235 | 1.7 | 202 | 1.3 | 0 |
| PNAPM | 2014 | 2014 | LA BOCANA | 5 | 15 | 212 | 9.4 | 199 | 9.0 | 0 |
| PNAPM | 2014 | 2014 | La Pared | 5 | 10 | 115 | 1.7 | 108 | 1.7 | 0 |
| PNAPM | 2014 | 2014 | Limones | 5 | 9 | 129 | 19.4 | 57 | 6.2 | 0 |
| HRI | 2014 | 2014 | Mx-1042 | 2 | 9 | 36 | 2.8 | 24 | 2.8 | 0 |
| HRI | 2014 | 2014 | Mx-1043 | 1 | 8 | 44 | 0.0 | 16 | 0.0 | 0 |
| HRI | 2014 | 2014 | Mx-1048 | 1 | 8 | 34 | 0.0 | 4 | 0.0 | 0 |
| HRI | 2014 | 2014 | Mx-1050 | 1 | 7 | 19 | 0.0 | 3 | 0.0 | 0 |
| HRI | 2014 | 2014 | Mx-1057 | 1 | 6 | 18 | 5.6 | 5 | 5.6 | 0 |
| HRI | 2014 | 2014 | Mx-1117 | 2 | 4 | 27 | 3.7 | 2 | 0.0 | 0 |
| HRI | 2014 | 2014 | Mx-1133 | 2 | 4 | 20 | 0.0 | 6 | 0.0 | 0 |
| PNAC | 2014 | 2014 | Paraiso | 6 | 10 | 48 | 0.0 | 14 | 0.0 | 0 |
| PNAC | 2014 | 2014 | Paso del Cedral | 6 | 11 | 42 | 0.0 | 10 | 0.0 | 0 |
| PNAPM | 2014 | 2014 | Radio Pirata | 5 | 10 | 117 | 8.5 | 85 | 2.6 | 0 |
| PNAPM | 2014 | 2014 | Tanchacte Norte | 5 | 12 | 150 | 5.3 | 101 | 1.3 | 0 |
| PNAPM | 2014 | 2014 | TANCHACTE SUR | 5 | 10 | 93 | 4.3 | 86 | 4.3 | 0 |
| PNAC | 2014 | 2014 | Yucab | 6 | 8 | 23 | 0.0 | 8 | 0.0 | 0 |
| Barco-Lab | 2016 | 2016 | Mx-1005 | 3 | 17 | 70 | 2.9 | 26 | 2.9 | 0 |
| Barco-Lab | 2016 | 2016 | Mx-1006 | 4 | 13 | 78 | 3.8 | 36 | 3.8 | 0 |
| Barco-Lab | 2016 | 2016 | Mx-1010 | 2 | 16 | 61 | 0.0 | 22 | 0.0 | 0 |
| Barco-Lab | 2016 | 2016 | Mx-1020 | 2 | 13 | 66 | 1.5 | 20 | 1.5 | 0 |
| Barco-Lab | 2016 | 2016 | Mx-1026 | 2 | 14 | 99 | 0.0 | 35 | 0.0 | 0 |
| Barco-Lab | 2016 | 2016 | Mx-1028 | 2 | 18 | 86 | 0.0 | 25 | 0.0 | 0 |
| Barco-Lab | 2016 | 2016 | Mx-1034 | 4 | 14 | 92 | 2.2 | 34 | 1.1 | 0 |
| Barco-Lab | 2016 | 2016 | Mx-1035 | 4 | 11 | 60 | 1.7 | 23 | 1.7 | 0 |
| Barco-Lab | 2016 | 2016 | Mx-1037 | 3 | 17 | 74 | 4.1 | 26 | 4.1 | 0 |
| Barco-Lab | 2016 | 2016 | Mx-1047 | 2 | 15 | 101 | 0.0 | 41 | 0.0 | 0 |
| Barco-Lab | 2016 | 2016 | Mx-1062 | 2 | 14 | 59 | 0.0 | 18 | 0.0 | 0 |
| Barco-Lab | 2016 | 2016 | Mx-1065 | 2 | 15 | 88 | 0.0 | 36 | 0.0 | 0 |
| Barco-Lab | 2016 | 2016 | Mx-1066 | 2 | 4 | 8 | 0.0 | 3 | 0.0 | 0 |
| Barco-Lab | 2016 | 2016 | Mx-1109 | 4 | 11 | 92 | 0.0 | 14 | 0.0 | 0 |
| Barco-Lab | 2016 | 2016 | Mx-1134 | 2 | 6 | 47 | 2.1 | 3 | 0.0 | 0 |
| Barco-Lab | 2016 | 2016 | Mx-2007 | 3 | 20 | 99 | 2.0 | 36 | 2.0 | 0 |
| Barco-Lab | 2016 | 2016 | Mx-2033 | 2 | 12 | 93 | 0.0 | 54 | 0.0 | 0 |
| Barco-Lab | 2016 | 2016 | Mx-3054 | 2 | 14 | 100 | 0.0 | 13 | 0.0 | 0 |
| Barco-Lab | 2016 | 2016 | Mx-1032B | 2 | 12 | 84 | 1.2 | 30 | 1.2 | 0 |
| Barco-Lab | 2016 | 2016 | Akumal Langosta | 2 | 17 | 103 | 3.9 | 36 | 3.9 | 0 |
| Barco-Lab | 2016 | 2016 | Bacalar Chico | 2 | 13 | 115 | 0.9 | 50 | 0.9 | 0 |
| Barco-Lab | 2016 | 2016 | Bonanza | 4 | 17 | 118 | 0.8 | 35 | 0.8 | 0 |
| Barco-Lab | 2016 | 2016 | Bonanza Profundo | 2 | 15 | 89 | 0.0 | 28 | 0.0 | 0 |
| Barco-Lab | 2016 | 2016 | Canones | 2 | 14 | 83 | 0.0 | 24 | 0.0 | 0 |
| Barco-Lab | 2016 | 2016 | Casa Cenote | 2 | 18 | 92 | 2.2 | 58 | 2.2 | 0 |
| Barco-Lab | 2016 | 2016 | Chankanaab | 2 | 12 | 108 | 2.8 | 31 | 0.9 | 0 |
| Barco-Lab | 2016 | 2016 | Chitales | 2 | 7 | 94 | 0.0 | 1 | 0.0 | 0 |
| Barco-Lab | 2016 | 2016 | Colombia | 2 | 17 | 104 | 1.0 | 20 | 0.0 | 0 |
| Barco-Lab | 2016 | 2016 | Colombia Profundo | 2 | 14 | 87 | 0.0 | 29 | 0.0 | 0 |
| Barco-Lab | 2016 | 2016 | Cordilleras | 2 | 12 | 80 | 1.3 | 24 | 1.3 | 0 |
| Barco-Lab | 2016 | 2016 | Cuevones | 2 | 10 | 96 | 0.0 | 6 | 0.0 | 0 |
| Barco-Lab | 2016 | 2016 | Cuevones Somero | 4 | 9 | 71 | 0.0 | 27 | 0.0 | 0 |
| Barco-Lab | 2016 | 2016 | Dalila | 2 | 15 | 101 | 1.0 | 20 | 1.0 | 0 |
| Barco-Lab | 2016 | 2016 | El Placer | 2 | 12 | 74 | 1.4 | 26 | 1.4 | 0 |
| Barco-Lab | 2016 | 2016 | Estufa | 4 | 23 | 209 | 0.0 | 62 | 0.0 | 0 |
| Barco-Lab | 2016 | 2016 | Fish Market | 1 | 9 | 41 | 0.0 | 6 | 0.0 | 0 |
| Barco-Lab | 2016 | 2016 | Hanan | 2 | 9 | 163 | 0.0 | 10 | 0.0 | 0 |
| Barco-Lab | 2016 | 2016 | Hanan II | 2 | 10 | 97 | 1.0 | 33 | 1.0 | 0 |
| Barco-Lab | 2016 | 2016 | Ixlaché | 2 | 9 | 87 | 0.0 | 11 | 0.0 | 0 |
| Barco-Lab | 2016 | 2016 | Jardines | 4 | 10 | 90 | 1.1 | 23 | 0.0 | 0 |
| Barco-Lab | 2016 | 2016 | LA BOCANA | 4 | 12 | 75 | 0.0 | 39 | 0.0 | 0 |
| Barco-Lab | 2016 | 2016 | La Pared | 4 | 14 | 112 | 0.0 | 26 | 0.0 | 0 |
| Barco-Lab | 2016 | 2016 | La Poza | 2 | 15 | 100 | 0.0 | 41 | 0.0 | 0 |
| Barco-Lab | 2016 | 2016 | Limones | 4 | 9 | 87 | 6.9 | 11 | 0.0 | 0 |
| Barco-Lab | 2016 | 2016 | Mah01 | 2 | 18 | 91 | 0.0 | 25 | 0.0 | 0 |
| Barco-Lab | 2016 | 2016 | Mahahual | 2 | 16 | 111 | 0.0 | 32 | 0.0 | 0 |
| Barco-Lab | 2016 | 2016 | Mahahual Centro | 2 | 16 | 109 | 0.0 | 48 | 0.0 | 0 |
| Barco-Lab | 2016 | 2016 | Mahahual1 | 2 | 17 | 95 | 0.0 | 29 | 0.0 | 0 |
| Barco-Lab | 2016 | 2016 | Manchones Norte | 2 | 13 | 50 | 0.0 | 30 | 0.0 | 0 |
| Barco-Lab | 2016 | 2016 | Mosquitero | 2 | 17 | 87 | 0.0 | 23 | 0.0 | 0 |
| Barco-Lab | 2016 | 2016 | MS | 1 | 9 | 38 | 0.0 | 7 | 0.0 | 0 |
| HRI | 2016 | 2016 | Mx-1017 | 3 | 7 | 112 | 0.0 | 16 | 0.0 | 0 |
| HRI | 2016 | 2016 | Mx-1048 | 2 | 10 | 39 | 0.0 | 9 | 0.0 | 0 |
| HRI | 2016 | 2016 | Mx-1053 | 3 | 13 | 71 | 0.0 | 26 | 0.0 | 0 |
| HRI | 2016 | 2016 | Mx-1133 | 4 | 11 | 46 | 0.0 | 18 | 0.0 | 0 |
| HRI | 2016 | 2016 | Mx-3009 | 2 | 12 | 76 | 0.0 | 6 | 0.0 | 0 |
| HRI | 2016 | 2016 | Mx-CHAN01 | 2 | 11 | 38 | 0.0 | 17 | 0.0 | 0 |
| HRI | 2016 | 2016 | Mx-COCO02 | 2 | 16 | 105 | 0.0 | 16 | 0.0 | 0 |
| HRI | 2016 | 2016 | Mx-PAR01 | 1 | 6 | 23 | 0.0 | 3 | 0.0 | 0 |
| HRI | 2016 | 2016 | Mx-TACU01 | 1 | 9 | 31 | 0.0 | 11 | 0.0 | 0 |
| Barco-Lab | 2016 | 2016 | Paraiso | 2 | 14 | 81 | 3.7 | 37 | 3.7 | 0 |
| Barco-Lab | 2016 | 2016 | Paso del Cedral | 2 | 16 | 80 | 0.0 | 22 | 0.0 | 0 |
| Barco-Lab | 2016 | 2016 | Punta Maroma Norte | 2 | 18 | 83 | 0.0 | 45 | 0.0 | 0 |
| Barco-Lab | 2016 | 2016 | Punta Maroma Sur | 2 | 15 | 72 | 0.0 | 33 | 0.0 | 0 |
| Barco-Lab | 2016 | 2016 | Punta Sur Mera Somero | 2 | 10 | 57 | 3.5 | 12 | 3.5 | 0 |
| Barco-Lab | 2016 | 2016 | Radio Pirata | 4 | 10 | 95 | 0.0 | 22 | 0.0 | 0 |
| Barco-Lab | 2016 | 2016 | San Clemente | 3 | 13 | 85 | 0.0 | 25 | 0.0 | 0 |
| Barco-Lab | 2016 | 2016 | Tampalam Centro | 2 | 17 | 81 | 1.2 | 39 | 1.2 | 0 |
| Barco-Lab | 2016 | 2016 | Tampalam Sur | 2 | 14 | 81 | 1.2 | 26 | 1.2 | 0 |
| Barco-Lab | 2016 | 2016 | Tanchacte | 2 | 14 | 71 | 1.4 | 38 | 0.0 | 0 |
| Barco-Lab | 2016 | 2016 | Tanchacte Norte | 4 | 14 | 134 | 0.0 | 50 | 0.0 | 0 |
| Barco-Lab | 2016 | 2016 | TANCHACTE SUR | 4 | 10 | 92 | 0.0 | 31 | 0.0 | 0 |
| Barco-Lab | 2016 | 2016 | Tormentos | 1 | 6 | 25 | 0.0 | 10 | 0.0 | 0 |
| Barco-Lab | 2016 | 2016 | Xahuayxol Norte 1 | 2 | 20 | 128 | 0.0 | 45 | 0.0 | 0 |
| Barco-Lab | 2016 | 2016 | XM04 | 2 | 11 | 41 | 0.0 | 12 | 0.0 | 0 |
| Barco-Lab | 2016 | 2016 | XYSF1 | 2 | 18 | 113 | 0.0 | 50 | 0.0 | 0 |
| Barco-Lab | 2016 | 2016 | Yucab | 2 | 14 | 83 | 0.0 | 25 | 0.0 | 0 |
| Barco-Lab | 2017 | 2017 | Mx-1014 | 3 | 16 | 94 | 0.0 | 18 | 0.0 | 0 |
| Barco-Lab | 2017 | 2017 | Mx-2052 | 3 | 18 | 153 | 0.7 | 51 | 0.0 | 0 |
| Barco-Lab | 2017 | 2017 | BAK | 1 | 28 | 208 | 0.0 | 83 | 0.0 | 0 |
| Barco-Lab | 2017 | 2017 | BBC | 1 | 12 | 200 | 0.0 | 95 | 0.0 | 0 |
| Barco-Lab | 2017 | 2017 | BP | 1 | 27 | 202 | 0.0 | 89 | 0.0 | 0 |
| Barco-Lab | 2017 | 2017 | Chankanaab | 6 | 15 | 141 | 0.7 | 39 | 0.7 | 0 |
| Barco-Lab | 2017 | 2017 | Colombia | 6 | 16 | 118 | 0.0 | 23 | 0.0 | 0 |
| Barco-Lab | 2017 | 2017 | Dalila | 6 | 16 | 130 | 0.0 | 10 | 0.0 | 0 |
| Barco-Lab | 2017 | 2017 | ESC | 1 | 30 | 208 | 0.0 | 110 | 0.0 | 0 |
| Barco-Lab | 2017 | 2017 | HMB | 1 | 24 | 205 | 0.0 | 76 | 0.0 | 0 |
| Barco-Lab | 2017 | 2017 | Jardines | 1 | 14 | 225 | 0.9 | 63 | 0.4 | 0 |
| Barco-Lab | 2017 | 2017 | LA BOCANA | 1 | 18 | 219 | 3.2 | 109 | 2.7 | 0 |
| Barco-Lab | 2017 | 2017 | Limones | 1 | 14 | 220 | 6.4 | 65 | 3.2 | 2 |
| Barco-Lab | 2017 | 2017 | P. Paila | 3 | 15 | 149 | 2.7 | 68 | 2.7 | 0 |
| Barco-Lab | 2017 | 2017 | P. Yuyum | 3 | 21 | 114 | 2.6 | 53 | 2.6 | 0 |
| Barco-Lab | 2017 | 2017 | Palancar Herradura | 2 | 14 | 107 | 0.0 | 11 | 0.0 | 0 |
| Barco-Lab | 2017 | 2017 | Paraiso | 6 | 16 | 76 | 0.0 | 35 | 0.0 | 0 |
| Barco-Lab | 2017 | 2017 | Paraiso Puerto Aventuras | 3 | 17 | 126 | 0.8 | 29 | 0.0 | 0 |
| Barco-Lab | 2017 | 2017 | Parche 10 | 1 | 19 | 201 | 0.0 | 57 | 0.0 | 0 |
| Barco-Lab | 2017 | 2017 | Parche 7 | 1 | 11 | 200 | 0.0 | 49 | 0.0 | 0 |
| Barco-Lab | 2017 | 2017 | Parche 9 | 1 | 11 | 200 | 0.0 | 32 | 0.0 | 0 |
| Barco-Lab | 2017 | 2017 | Paso del Cedral | 6 | 9 | 70 | 0.0 | 16 | 0.0 | 0 |
| Barco-Lab | 2017 | 2017 | PJ10 | 1 | 14 | 200 | 0.0 | 102 | 0.0 | 0 |
| Barco-Lab | 2017 | 2017 | PL10 | 1 | 18 | 200 | 0.0 | 96 | 0.0 | 0 |
| Barco-Lab | 2017 | 2017 | PP10 | 1 | 12 | 200 | 0.0 | 103 | 0.0 | 0 |
| Barco-Lab | 2017 | 2017 | Radio Pirata | 1 | 21 | 219 | 0.0 | 114 | 0.0 | 0 |
| Barco-Lab | 2017 | 2017 | SIR | 1 | 25 | 206 | 0.0 | 78 | 0.0 | 0 |
| Barco-Lab | 2017 | 2017 | SMDR10 | 1 | 14 | 200 | 0.0 | 114 | 0.0 | 0 |
| Barco-Lab | 2017 | 2017 | TANCHACTE SUR | 1 | 23 | 211 | 3.8 | 100 | 2.8 | 0 |
| Barco-Lab | 2017 | 2017 | Yucab | 6 | 11 | 92 | 1.1 | 30 | 1.1 | 0 |
| Barco-Lab | 2018 | 2018/2019 | Bonanza | 5 | 11 | 86 | 1.2 | 17 | 1.2 | 0 |
| Barco-Lab | 2018 | 2018/2019 | Colombia | 1 | 24 | 376 | 4.3 | 112 | 2.9 | 2 |
| Barco-Lab | 2018 | 2018/2019 | Colombia | 1 | 26 | 353 | 15.9 | 112 | 3.1 | 2 |
| Barco-Lab | 2018 | 2018/2019 | Dicks | 5 | 18 | 183 | 6.6 | 94 | 6.0 | 0 |
| Barco-Lab | 2018 | 2018/2019 | Fish Market | 14 | 22 | 825 | 18.3 | 349 | 17.2 | 31 |
| Barco-Lab | 2018 | 2018/2019 | Fish Market | 16 | 18 | 333 | 7.5 | 111 | 4.8 | 57 |
| Barco-Lab | 2018 | 2018/2019 | Jardines | 6 | 14 | 206 | 8.7 | 103 | 7.3 | 0 |
| Barco-Lab | 2018 | 2018/2019 | Jardines Turistico | 4 | 19 | 410 | 8.8 | 94 | 8.3 | 4 |
| Barco-Lab | 2018 | 2018/2019 | LA BOCANA | 15 | 19 | 232 | 8.6 | 132 | 8.2 | 0 |
| Barco-Lab | 2018 | 2018/2019 | La Pared | 9 | 17 | 284 | 2.1 | 149 | 1.4 | 0 |
| Barco-Lab | 2018 | 2018/2019 | Limones | 22 | 20 | 396 | 9.8 | 64 | 5.6 | 0 |
| Barco-Lab | 2018 | 2018/2019 | Limones | 23 | 13 | 474 | 8.6 | 87 | 7.4 | 23 |
| Barco-Lab | 2018 | 2018/2019 | Manchones Norte | 9 | 25 | 390 | 13.1 | 170 | 11.0 | 1 |
| Barco-Lab | 2018 | 2018/2019 | Manchones Norte | 1 | 11 | 33 | 21.2 | 18 | 18.1 | 1 |
| Barco-Lab | 2018 | 2018/2019 | Mar F2 | 1 | 8 | 43 | 7.0 | 6 | 4.7 | 0 |
| Barco-Lab | 2018 | 2018/2019 | Mar F4 | 2 | 7 | 72 | 16.7 | 28 | 9.7 | 4 |
| Barco-Lab | 2018 | 2018/2019 | Media Luna | 5 | 22 | 205 | 12.7 | 87 | 6.3 | 2 |
| HRI | 2018 | 2018/2019 | Mx-1008 | 2 | 9 | 51 | 2.0 | 14 | 2.0 | 1 |
| HRI | 2018 | 2018/2019 | Mx-1014 | 2 | 18 | 149 | 3.4 | 29 | 1.3 | 0 |
| HRI | 2018 | 2018/2019 | Mx-1017 | 1 | 5 | 60 | 0.0 | 3 | 0.0 | 0 |
| HRI | 2018 | 2018/2019 | Mx-1020 | 2 | 17 | 110 | 3.6 | 22 | 1.8 | 3 |
| HRI | 2018 | 2018/2019 | Mx-1042 | 2 | 7 | 32 | 0.0 | 9 | 0.0 | 0 |
| HRI | 2018 | 2018/2019 | Mx-1043 | 1 | 12 | 77 | 1.3 | 40 | 1.3 | 0 |
| HRI | 2018 | 2018/2019 | Mx-1050 | 2 | 13 | 58 | 1.7 | 21 | 1.7 | 0 |
| HRI | 2018 | 2018/2019 | Mx-1055 | 1 | 5 | 10 | 0.0 | 7 | 0.0 | 0 |
| HRI | 2018 | 2018/2019 | Mx-1057 | 2 | 9 | 67 | 4.5 | 5 | 0.0 | 0 |
| HRI | 2018 | 2018/2019 | Mx-1065 | 2 | 23 | 164 | 0.6 | 44 | 0.6 | 0 |
| HRI | 2018 | 2018/2019 | Mx-1075 | 6 | 18 | 222 | 0.0 | 57 | 0.0 | 0 |
| HRI | 2018 | 2018/2019 | Mx-1116 | 2 | 5 | 20 | 0.0 | 2 | 0.0 | 0 |
| HRI | 2019 | 2018/2019 | Mx-1117 | 2 | 11 | 62 | 0.0 | 20 | 0.0 | 0 |
| HRI | 2019 | 2018/2019 | Mx-1125 | 2 | 7 | 166 | 3.6 | 2 | 0.0 | 0 |
| HRI | 2019 | 2018/2019 | Mx-1131 | 2 | 15 | 69 | 2.9 | 19 | 1.4 | 0 |
| HRI | 2018 | 2018/2019 | Mx-1132 | 1 | 7 | 45 | 8.9 | 0 | 0.0 | 0 |
| HRI | 2018 | 2018/2019 | Mx-1133 | 2 | 8 | 18 | 0.0 | 6 | 0.0 | 0 |
| HRI | 2018 | 2018/2019 | Mx-1136 | 2 | 5 | 42 | 0.0 | 5 | 0.0 | 0 |
| HRI | 2018 | 2018/2019 | Mx-2009 | 6 | 20 | 362 | 0.6 | 29 | 0.3 | 0 |
| HRI | 2018 | 2018/2019 | Mx-2052 | 1 | 14 | 109 | 5.5 | 21 | 3.7 | 0 |
| HRI | 2018 | 2018/2019 | Mx-2067 | 2 | 17 | 165 | 1.8 | 72 | 1.8 | 0 |
| HRI | 2018 | 2018/2019 | Mx-2078 | 6 | 21 | 272 | 2.2 | 130 | 2.2 | 2 |
| HRI | 2018 | 2018/2019 | Mx-2080 | 6 | 19 | 228 | 0.0 | 87 | 0.0 | 0 |
| HRI | 2018 | 2018/2019 | Mx-3054 | 4 | 11 | 236 | 3.0 | 29 | 1.7 | 6 |
| HRI | 2018 | 2018/2019 | Mx-3082 | 6 | 23 | 268 | 0.7 | 113 | 0.7 | 0 |
| HRI | 2018 | 2018/2019 | Mx-4160 | 6 | 15 | 241 | 5.4 | 27 | 0.8 | 2 |
| HRI | 2018 | 2018/2019 | Mx-CHI01 | 6 | 20 | 194 | 0.5 | 97 | 0.5 | 0 |
| HRI | 2018 | 2018/2019 | Mx-CHI02 | 6 | 16 | 264 | 1.1 | 78 | 0.0 | 2 |
| HRI | 2018 | 2018/2019 | Mx-CHI03 | 6 | 15 | 252 | 1.2 | 94 | 1.2 | 0 |
| HRI | 2018 | 2018/2019 | Mx-CHI04 | 6 | 20 | 240 | 0.8 | 81 | 0.4 | 0 |
| HRI | 2018 | 2018/2019 | Mx-CHI05 | 5 | 14 | 211 | 0.5 | 27 | 0.0 | 0 |
| HRI | 2018 | 2018/2019 | Mx-CUEV18 | 1 | 7 | 57 | 0.0 | 6 | 0.0 | 0 |
| HRI | 2018 | 2018/2019 | Mx-HANA02 | 4 | 12 | 254 | 6.3 | 34 | 3.9 | 6 |
| HRI | 2018 | 2018/2019 | Mx-HANAII | 7 | 18 | 236 | 11.0 | 74 | 8.5 | 9 |
| HRI | 2018 | 2018/2019 | Mx-ISLO01 | 6 | 16 | 262 | 0.0 | 57 | 0.0 | 0 |
| HRI | 2018 | 2018/2019 | Mx-Mah01 | 2 | 19 | 122 | 0.8 | 27 | 0.8 | 1 |
| HRI | 2018 | 2018/2019 | Mx-TACU01 | 2 | 19 | 120 | 2.5 | 73 | 2.5 | 1 |
| HRI | 2018 | 2018/2019 | Mx-XCK01 | 2 | 20 | 183 | 1.6 | 59 | 1.6 | 0 |
| HRI | 2018 | 2018/2019 | Mx-XCK02 | 2 | 19 | 156 | 1.3 | 41 | 0.0 | 0 |
| Barco-Lab | 2018 | 2018/2019 | Niccehabin 6m | 5 | 15 | 178 | 17.4 | 38 | 11.8 | 4 |
| Barco-Lab | 2018 | 2018/2019 | Niccehabin Frontal Somero | 6 | 21 | 270 | 13.7 | 70 | 10.4 | 2 |
| Barco-Lab | 2018 | 2018/2019 | Niccehabin Posterior | 4 | 14 | 196 | 4.6 | 21 | 3.6 | 0 |
| Barco-Lab | 2018 | 2018/2019 | Nizuc C3 | 2 | 7 | 43 | 16.3 | 4 | 7.0 | 1 |
| Barco-Lab | 2018 | 2018/2019 | Nizuc F3 | 7 | 11 | 35 | 28.6 | 14 | 11.4 | 6 |
| Barco-Lab | 2018 | 2018/2019 | Paraiso | 1 | 22 | 373 | 29.2 | 202 | 26.0 | 34 |
| Barco-Lab | 2018 | 2018/2019 | Paraíso | 1 | 19 | 423 | 17.0 | 226 | 13.0 | 39 |
| Barco-Lab | 2018 | 2018/2019 | Paso del Cedral | 1 | 25 | 356 | 2.5 | 141 | 1.7 | 1 |
| Barco-Lab | 2018 | 2018/2019 | Paso del Cedral | 1 | 25 | 329 | 17.6 | 141 | 1.8 | 1 |
| Barco-Lab | 2018 | 2018/2019 | PM-F2 | 2 | 9 | 93 | 24.7 | 36 | 11.8 | 13 |
| Barco-Lab | 2018 | 2018/2019 | Punta Allen Centro | 4 | 21 | 192 | 13.5 | 56 | 9.4 | 5 |
| Barco-Lab | 2018 | 2018/2019 | Punta Allen Norte | 5 | 18 | 274 | 13.5 | 70 | 10.9 | 8 |
| Barco-Lab | 2018 | 2018/2019 | Punta Maroma Centro_Profundo | 3 | 15 | 292 | 9.6 | 105 | 7.2 | 2 |
| Barco-Lab | 2018 | 2018/2019 | Punta Maroma Norte Profundo | 2 | 16 | 184 | 13.6 | 71 | 13.6 | 3 |
| Barco-Lab | 2018 | 2018/2019 | Punta Maroma Sur_Medio | 3 | 13 | 145 | 2.1 | 33 | 1.4 | 0 |
| Barco-Lab | 2019 | 2018/2019 | Punta Maroma Sur_Profundo | 3 | 14 | 184 | 8.7 | 62 | 6.5 | 2 |
| Barco-Lab | 2019 | 2018/2019 | Punta Maroma_Norte Medio | 3 | 13 | 242 | 1.2 | 37 | 1.2 | 0 |
| Barco-Lab | 2019 | 2018/2019 | Punta Maroma_Norte Somero | 3 | 13 | 294 | 1.0 | 14 | 1.0 | 0 |
| Barco-Lab | 2019 | 2018/2019 | Radio Pirata | 8 | 17 | 200 | 6.0 | 46 | 5.0 | 1 |
| Barco-Lab | 2019 | 2018/2019 | San Antonio 6m | 5 | 18 | 330 | 13.3 | 86 | 7.9 | 6 |
| Barco-Lab | 2018 | 2018/2019 | San Antonio Posterior | 6 | 13 | 244 | 7.4 | 16 | 0.4 | 0 |
| Barco-Lab | 2018 | 2018/2019 | Tanchacte | 14 | 25 | 544 | 12.3 | 293 | 10.7 | 8 |
| Barco-Lab | 2018 | 2018/2019 | Yal Ku | 5 | 24 | 229 | 15.7 | 69 | 8.3 | 2 |
| Barco-Lab | 2018 | 2018/2019 | Yuyum 6m | 6 | 9 | 123 | 5.7 | 16 | 4.9 | 0 |
| Barco-Lab | 2018 | 2018/2019 | Yuyum Frontal Somero | 4 | 15 | 202 | 15.8 | 37 | 7.4 | 2 |
| Barco-Lab | 2018 | 2018/2019 | Yuyum Posterior | 5 | 9 | 166 | 16.3 | 34 | 7.8 | 0 |
