# Supplementary material for: A rapid spread of the stony coral tissue loss disease outbreak in the Mexican Caribbean
Source: PeerJ. 2019 Nov 26;7:e8069. doi: 10.7717/peerj.8069 (PMC6883952; doi:10.7717/peerj.8069)
Supplement: Supplemental Information 3 — Death colonies are only those for which death could be attributable to the SCTLD (exposed bright white skeletons; see Fig. 1). [file peerj-07-8069-s003.docx]

Table S2. Total number of colonies recorded for each species across the 82 surveyed reefs in the Mexican Caribbean (2018 and 2019). Death colonies are only those for which death could be attributable to the SCTLD (exposed bright white skeletons; see Fig. 1).

| Species | Total number of colonies | Afflicted colonies by the SCTLD | Number of recently dead colonies |
| --- | --- | --- | --- |
| *Dendrogyra cylindrus* | 7 | 5 | 0 |
| *Siderastrea siderea* | 2367 | 545 | 72 |
| *Pseudodiploria strigosa* | 678 | 150 | 125 |
| *Eusmilia fastigiata* | 158 | 31 | 22 |
| *Colpophyllia natans* | 75 | 14 | 2 |
| *Montastraea cavernosa* | 857 | 151 | 42 |
| *Diploria labyrinthiformis* | 88 | 15 | 7 |
| *Orbicella faveolata* | 443 | 70 | 2 |
| *Dichocoenia stokesii* | 61 | 7 | 4 |
| *Orbicella annularis* | 292 | 31 | 0 |
| *Meandrina meandrites* | 222 | 19 | 66 |
| *Agaricia lamarcki* | 42 | 3 | 0 |
| *Mycetophyllia sp.* | 36 | 2 | 4 |
| *Orbicella franksi* | 54 | 3 | 2 |
| *Favia fragum* | 57 | 3 | 4 |
| *Agaricia agaricites* | 4385 | 197 | 19 |
| *Helioseris cucullata* | 68 | 3 | 0 |
| *Stephanocoenia intersepta* | 332 | 12 | 1 |
| *Isophyllia rigida* | 34 | 1 | 2 |
| *Agaricia tenuifolia* | 517 | 13 | 0 |
| *Porites astreoides* | 3606 | 73 | 14 |
| *Agaricia humilis* | 85 | 1 | 0 |
| *Porites furcata* | 98 | 1 | 1 |
| *Porites porites* | 874 | 6 | 0 |
| *Agaricia fragilis* | 11 | 0 | 0 |
| *Agaricia undata* | 3 | 0 | 0 |
| *Especie desconocida* | 1 | 0 | 0 |
| *Isophyllia sinuosa* | 13 | 0 | 0 |
| *Isophyllia sp.* | 5 | 0 | 0 |
| *Madracis auretenra* | 24 | 0 | 0 |
| *Madracis decactis* | 75 | 0 | 0 |
| *Manicina areolata* | 6 | 0 | 0 |
| *Mussa angulosa* | 1 | 0 | 0 |
| *Mycetophyllia aliciae* | 4 | 0 | 0 |
| *Mycetophyllia daniana* | 2 | 0 | 0 |
| *Mycetophyllia ferox* | 1 | 0 | 0 |
| *Mycetophyllia lamarckiana* | 25 | 0 | 0 |
| *Porites divaricata* | 143 | 0 | 0 |
| *Pseudodiploria clivosa* | 32 | 0 | 1 |
| *Scolymia sp.* | 8 | 0 | 0 |
| *Solenastrea bournoni* | 5 | 0 | 0 |
